# *miRNA-221-3p* Enhances the Secretion of Interleukin-4 in Mast Cells through the Phosphatase and Tensin Homolog/p38/Nuclear Factor-kappaB Pathway

**DOI:** 10.1371/journal.pone.0148821

**Published:** 2016-02-22

**Authors:** Yao Zhou, Qianyuan Yang, Hong Xu, Jiamin Zhang, Huan Deng, Haiyan Gao, Jin Yang, Deyu Zhao, Feng Liu

**Affiliations:** 1 Department of Respiratory Medicine, Nanjing Children's Hospital Affiliated to Nanjing Medical University, Nanjing, Jiangsu, China 210008; 2 Department of Pediatrics, Jiangsu Huai'an Maternity and Children's Hospital, Huai'an, Jiangsu, China 223002; National University of Singapore, SINGAPORE

## Abstract

Mast cells play a central role in asthma. Moreover, serum *miRNA-221-3p* (*miR-221*) has been shown to be markedly increased in children with asthma. In the current study, we aimed to examine *miR-221* expression in an asthma model and elucidate the mechanisms regulating interleukin (IL)-4 secretion in mast cells. Using polymerase chain reaction, we found that *miR-221* was upregulated in a murine asthma model and in P815 mast cells after lipopolysaccharide (LPS) stimulation. Moreover, *miR-221* upregulated IL-4 secretion from P815 cells, as shown by enzyme-linked immunosorbent assays. Bioinformatics analysis, luciferase reporter gene assays, and western blotting showed that phosphatase and tensin homolog (PTEN) was a target of *miR-221* and could block IL-4 secretion stimulated by *miR-221*. The phosphorylation of p38 (protein) and activity of nuclear factor-kappaB (NF-κB) were increased after overexpression of *miR-221*, as shown by electrophoretic mobility shift assays. Finally, treatment with specific inhibitors could block IL-4 secretion. In conclusion, *miR-221*, which was overexpressed in a murine asthma model, stimulated IL-4 secretion in mast cells through a pathway involving PTEN, p38, and NF-κB.

## Introduction

Bronchial asthma is caused by chronic inflammation of the airways involving a variety of cells, including eosinophils, mast cells, and their components. Asthma has the highest morbidity of all childhood chronic respiratory diseases and its prevalence has increased significantly [1]; however, the pathophysiology of asthma is still not completely clear.

Studies have shown that mast cells are critical for the immediate reaction in bronchial asthma [2]. By releasing a large number of inflammatory mediators, mast cells can regulate immunoglobulin E (IgE)-mediated reactions, cause bronchoconstriction, increase mucosal microvascular permeability, and induce airway mucosa edema. Moreover, mast cells secrete the inflammatory mediator interleukin 4 (IL-4), which plays an important role in bronchial asthma by activating B lymphocytes to produce IgE [3], inducing eosinophil infiltration in the airway [4], and promoting inflammatory cell chemotaxis. In addition, IL-4 contributes to airway hyper-responsiveness [5].

MicroRNAs (miRNAs) are small noncoding RNAs that are implicated in diverse biological processes and diseases. miRNAs regulate gene expression post-transcriptionally by targeting the 3′-untranslated region (UTR) of specific mRNAs for degradation or translational repression. Emerging evidence has supported that there is a link between miRNAs and bronchial asthma. Moreover, several miRNAs, including *miR-1248*, *miR-26a*, *Let-7a*, and *Let-7d*, have been shown to be altered in the serum of patients with asthma [6]. We have previously found miR-221 increased in serum of asthmatic children[7]. How the microRNA act in the development of asthma worth more studies.

In this study, we aimed to explore the role of *miR-221* in the regulation of mast cell secretion by investigating changes in *miR-221* in a murine lung asthma model. Additionally, we tested the hypothesis that *miR-221* regulates the secretory function of mouse mast cells, focusing on identification of *miR-221* target genes and signaling pathways.

## Materials and Methods

### Preparation of the asthma model and treatment of mice

Female BALB/c mice weighing approximately 25 g each (Laboratory Animal Center, Nanjing Medical University, Jiangsu, China) were randomly divided into two groups (n = 6). Animals were kept in a controlled environment and fed standard food pellets and water. After 1 week of acclimation, on days 0 and 14, the mice were injected intraperitoneally with either 20 μg ovalbumin (OVA; Sigma-Aldrich Corp., St. Louis, MO, USA) and 20 mg Al(OH)3 in 0.2 mL phosphate-buffered saline (PBS) or PBS only as the control. Following sensitization, the mice were exposed to either aerosolized 1.0% OVA/PBS or PBS only for 20 min once a day on days 27–30. On day 31, mice were anaesthetized with an intraperitoneal injection of 1% chloral hydrate (Sigma) as previously described [8], cell numbers in the bronchoalveolar lavage fluid (BALF) were counted, and the lungs were removed and kept at -70°C for RNA extraction. Basically, mice were euthanized by cervical dislocation under anesthesia. Actually, in this study mice died when the PBS was infused into the lungs to collect the BALF after anaesthetization. All mice were housed in a specific pathogen-free facility and treated in accordance with the guidelines of the Care and Use of Laboratory Animals of the Ministry of Health, China. The study was approved by the Nanjing Medical University Animal and Use Committee.

### Cell culture and infection

P815 cells from the Biochemistry and Cell Biology Institute of the Chinese Academy of Sciences (TCM12), Shanghai (in 2011) were cultured in Dulbecco’s modified Eagle’s medium (DMEM) supplemented with 10% fetal bovine serum (FBS; Gibco BRL, Carlsbad, CA, USA) at 37°C in a 5% CO_2_ incubator until reaching 20% confluence in six-well cell culture plates. The cells were then infected with lentiviral vector 3-mmu-*miR-221* (the *miR-221* group), lentiviral vector 3-normal control (the LV3NC group), or lentiviral vector 5-mmu-PTEN (the PTEN group), purchased from GenePharma (Shanghai, China). The sequence of mature *miR-221* (5′-AGCUACAUUGUCUGCUGGGUUUC-3′) was obtained from miRBase (http://www.mirbase.org/). The medium was refreshed 48 h after infection, and the cells were then treated with 1 μg/mL puromycin (Life Technologies, Carlsbad, CA, USA) for 48 h. *miR-221* inhibitor was synthesized by RiboBio Co., Ltd. (Guangzhou, China). For transfection, the cells were grown to 20% confluence and transfected with *miR-221* inhibitor (the *miR-221* inhibitor group) or inhibitor negative control using Lipofectamine 2000 (Lip 2000; Life Technologies) followed by incubation in Opti-Mem I for 6 h. The cells were then transferred into fresh DMEM containing 10% FBS. After incubating for 24 h, the culture medium was replaced for another 16 h.

To further explore the role of p38 in the regulation of *miR-221* in the Toll-like receptor (TLR) signaling pathway, cells transfected with lentiviral vector 3-mmu-*miR-221* were treated for 30 min with a specific p38 signaling pathway inhibitor, SB203580 (20 μmol/L), followed by stimulation with lipopolysaccharide (LPS) for 6 h. Alternatively, P815 cells transfected with control vector or lentiviral vector 3-mmu-*miR-221* were stimulated with LPS alone for 6 h. To determine the role of NF-κB in the regulation of *miR-221*, cells were treated with the NF-κB inhibitor ammonium pyrrolidinedithiocarbamate (PDTC) at a concentration of 50 μmol/L. All cells and supernatants were harvested for analysis.

### Luciferase reporter assay

P815 cells were seeded in 24-well culture plates the day before transfection. The cells were then cotransfected with a vector containing the PTEN-3′UTR and an *miR-221* mimic (Invitrogen, Carlsbad, CA, USA). At 48 h after transfection, the lysates were harvested, and the luciferase activities were measured using a Dual Luciferase Reporter Assay kit (Promega, Madison, WI, USA). All data were obtained by averaging the results from four independent repeats of the assay.

### Microarray analysis

To identify potential changes to signaling pathways related to disruptions in *miR-221* expression, cells transfected with the control vector, lentiviral vector 3-mmu-*miR-221*, or *miR-221* inhibitor were subjected to microarray analysis (KangChen Bio-tech Inc., Shanghai, China) as previously described [9].

### Measurement of cytokine production

The culture supernatants from both treated and untreated cells were collected as described above. IL-4 levels in the culture supernatants were measured for the different groups of cells using enzyme-linked immunosorbent assays (ELISAs; R&D Systems, Inc., MN, USA), according to the manufacturer’s recommendations.

### RNA extraction and quantitative polymerase chain reaction (PCR)

Total RNA was extracted from cells using TRIzol reagent (Invitrogen). All procedures for each assay were performed according to the manufacturer’s recommendations. The primers for *miR-221* and *RNU6* were purchased from Invitrogen, and those for *GAPDH* and *PTEN* were purchased from Sangon Biotech (Shanghai, China). PCR was performed using the 7500 Fast Real-Time PCR System (Life Technologies).

### Western blot analysis

Cells were lysed in ice-cold radio-immunoprecipitation assay buffer (Beyotime Institute of Biotechnology, Nantong, Jiangsu, China). The total protein concentration was determined using an Enhanced BCA Protein Assay Kit (Beyotime Institute of Biotechnology). Equal amounts of proteins were then separated by 10% sodium dodecyl sulfate polyacrylamide gel electrophoresis and transferred onto nitrocellulose filter membranes (Millipore, Billerica, MA, USA). After blocking in Tris-buffered saline (TBS) containing 5% nonfat milk, blots were incubated in blocking buffer (1× TBS containing 0.1% Tween-20 and 5% nonfat dry milk) for 2 h, followed by incubation with primary antibodies targeting GAPDH (sc-25778; isotype: rabbit IgG; 1:1000 dilution; ZSGB-Bio, Beijing, China), PTEN (9559s; isotype: rabbit IgG; 1:800 dilution; Cell Signaling Technology, Beverly, MA, USA), p38 mitogen-activated protein kinase (MAPK; 8690p; isotype: rabbit IgG; 1:2000 dilution; Cell Signaling Technology), and phospho-p38 MAPK (Thr180/Tyr182; 4511s; isotype: rabbit IgG; 1:1000 dilution; Cell Signaling Technology) at 4°C for 12 h. The membranes were then incubated with horseradish peroxidase-conjugated anti-rabbit or anti-mouse antibodies (ZDR-5306; isotype: goat IgG; 1:5000 dilution; ZSGB-Bio) at room temperature for 2 h. Signals were detected on a gel imaging system using ECL western blotting substrate (Thermo Fisher Scientific, MA, USA).

### Electrophoretic mobility shift assay (EMSA)

NF-κB protein was extracted from the nuclei of cells (Vazyme, NJ, USA) according to manufacturer’s instructions. EMSA was then carried out using a kit purchased from Pierce (Rockford, IL, USA) according to the manufacturer’s recommendations.

### Statistical analyses

All data were expressed as means ± standard errors (SEs). For the statistical analyses, comparisons were carried out using Student’s t-tests. Differences with *P* values of less than 0.05 were considered significant.

## Results

### *miR-221* was upregulated in a murine asthma model

**Fig 1:**
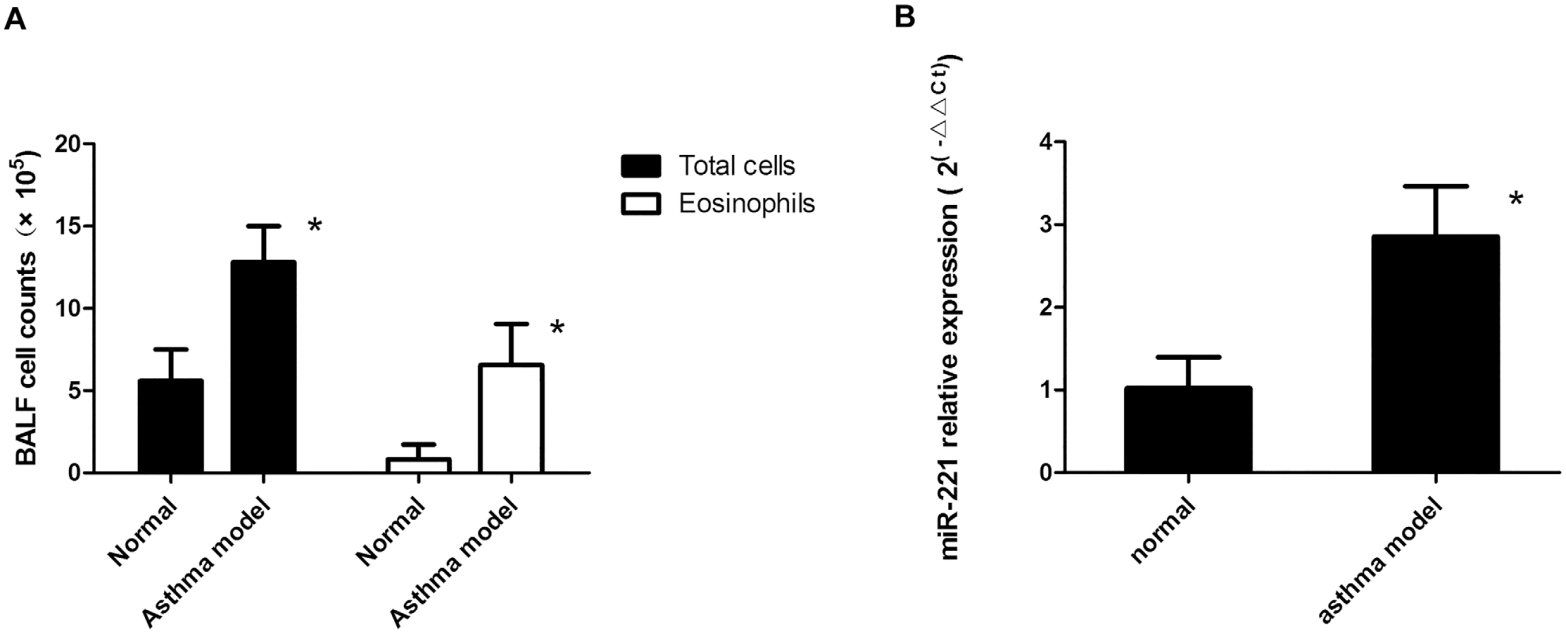
*miR-221* expression in a murine asthma model. A) Total cell and eosinophil numbers. B) Expression of *miR-221* in the murine asthma model. *: *P* < 0.05, compared with the normal group (n = 8).

In BALF from our murine asthma model, the numbers of total cells and eosinophils increased, indicating the presence of airway inflammation. Because *miR-221* was found to be increased in pediatric asthmatics [7], reverse transcription (RT)-PCR was used to confirm the changes in lung tissues in the murine asthma model. This analysis showed that *miR-221* was increased by approximately three-fold in this model (Fig 1A and 1B).

### *miR-221* was upregulated after treatment with LPS in mast cells and regulated the levels of IL-4 in the supernatant

**Fig 2:**
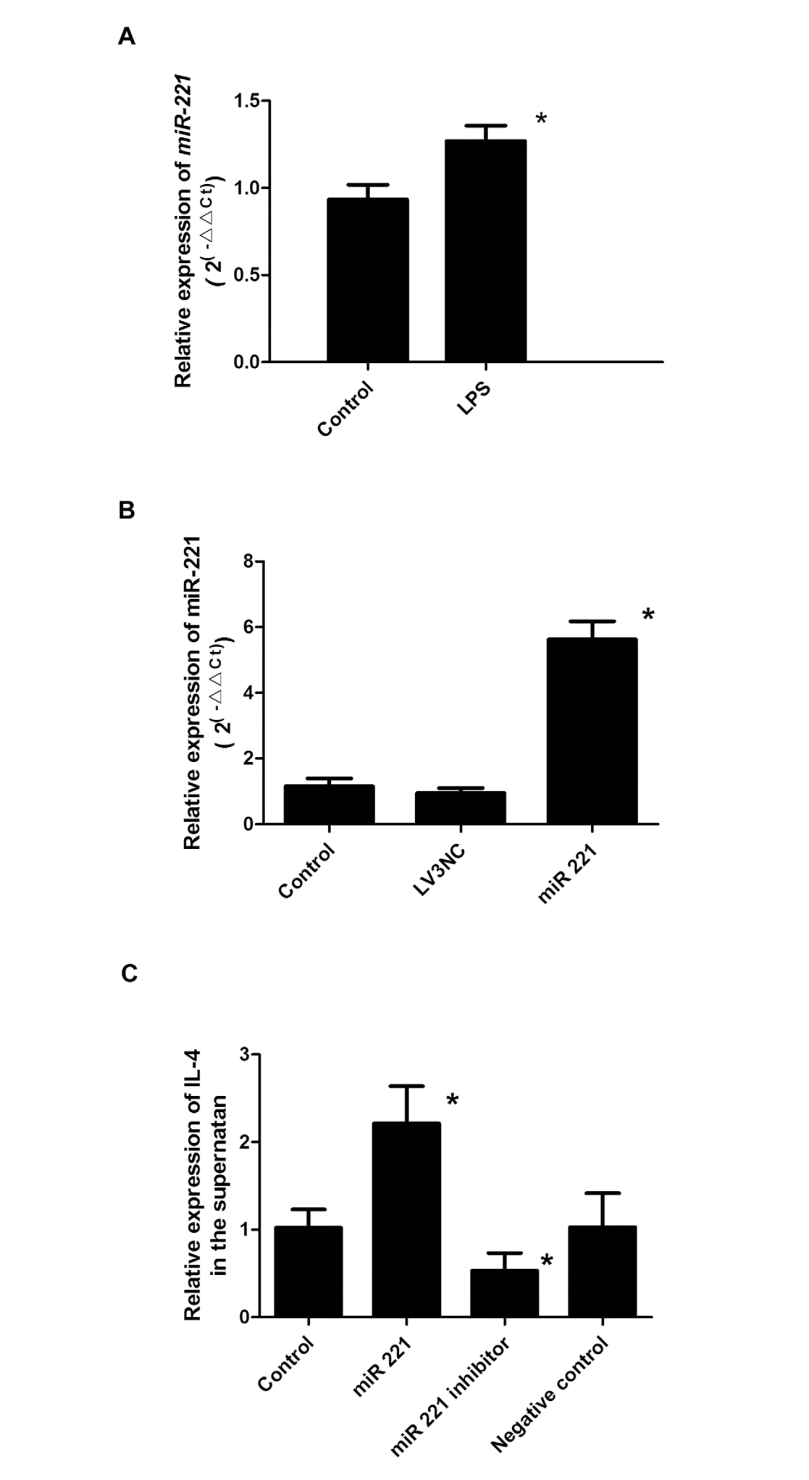
*miR-221* regulated IL-4 levels in the supernatants of P815 cells. A) Effects of LPS stimulation on the expression of *miR-221* in P815 cells. *: *P* < 0.05 (n = 6). B). P815 cells were transfected with an *miR-221* overexpression lentivirus vector, and *miR-221* expression was then assessed in the blank control group, negative virus group (LV3NC), and *miR-221*-transfected cells. *: *P* < 0.05 (n = 6). C) Enzyme-linked immunosorbent assays were used to assess IL-4 levels in the supernatants in the *miR-221*, blank control, and *miR-221* inhibitor (*miR-221*in) groups. *: *P* < 0.05 (n = 6). Negative control: cells transfected with inhibitor negative control.

Next, we analyzed the effects of LPS on *miR-221* expression. After a 16-h treatment with LPS, cells were collected, and *miR-221* was detected by RT-PCR. As shown in Fig 2A, cells treated with LPS exhibited higher levels of *miR-221* than control cells (*P* < 0.05).

To determine the role of *miR-221* in IL-4 secretion from P815 cells, gain- and loss-of-function experiments were conducted by transfecting P815 cells with a negative control lentivirus, a synthetic specific overexpression lentivirus, or a specific *miR-221* inhibitor. The expression levels of mature *miR-221* increased after transfection with the miRNA overexpression lentivirus but not with the negative control lentivirus, indicating successful transfection (*P* < 0.05; Fig 2B). Additionally, IL-4 levels in the supernatant increased after transfection with the *miR-221* overexpression lentivirus and decreased after transfection with the *miR-221* inhibitor. No differences were observed between the normal control group and negative control group (Fig 2C).

### PTEN was a target of *miR-221*

**Fig 3:**
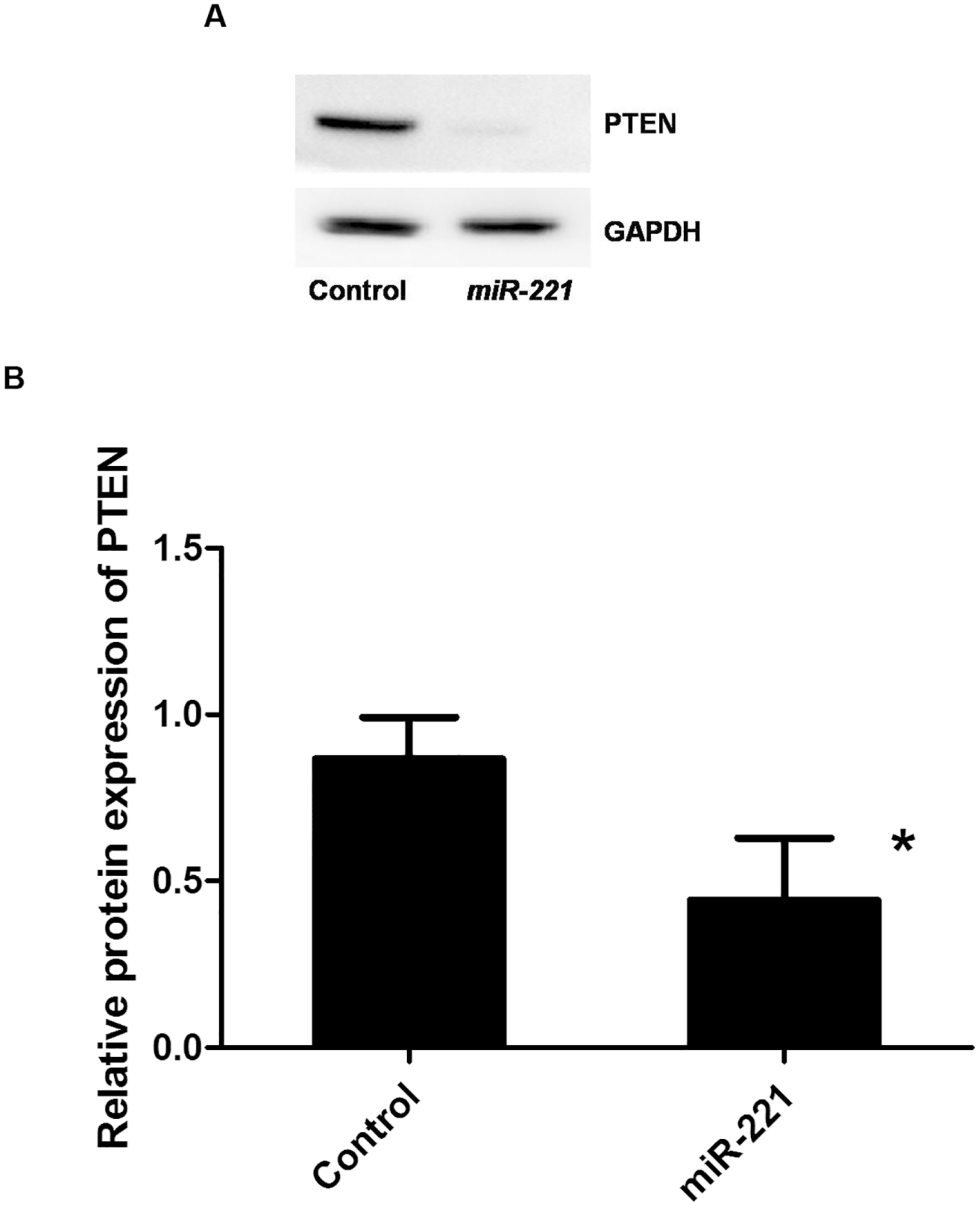
Effects of *miR-221* levels on PTEN protein expression. A, B) *miR-221* was overexpressed in cells, and PTEN expression was analyzed. *: *P* < 0.05 (n = 3 experiments).

By bioinformatics analysis, PTEN was predicted to be a target of *miR-221* at two different 3′-UTR sites. In addition, PTEN has recently been shown to have a role in allergic diseases. Therefore, in this study, PTEN was chosen for further research. Western blot analysis revealed that PTEN protein levels were decreased in cells overexpressing *miR-221* compared with those in normal cells (Fig 3A and 3B). A 3′-UTR reporter assay was then conducted to determine whether *miR-221* bound to the 3′-UTR of the mouse *PTEN* gene. The results showed that the luciferase activity of cells transfected with the *miR-221* overexpression vector was decreased compared with that in cells transfected with the blank vector control (mimic NC group), demonstrating that PTEN was inhibited by binding of *miR-221* to its 3′-UTR (Table 1).

**Table 1:** Dual luciferase assay on miR-221 and PTEN 3’-UTR (n = 3.)

	Gene (3′-UTR)	mean	SD	P value
mimics NC	PTEN1*	0.39	0.03	0.007
mmu-miR-221 mimics	PTEN1	0.31	<0.01	
mimics NC	PTEN2	0.56	0.01	0.002
mmu-miR-221 mimics	PTEN2	0.51	0.01	

*: two binding sites with miR-221.

### PTEN reversed the effects of *miR-221* on IL-4 secretion

**Fig 4:**
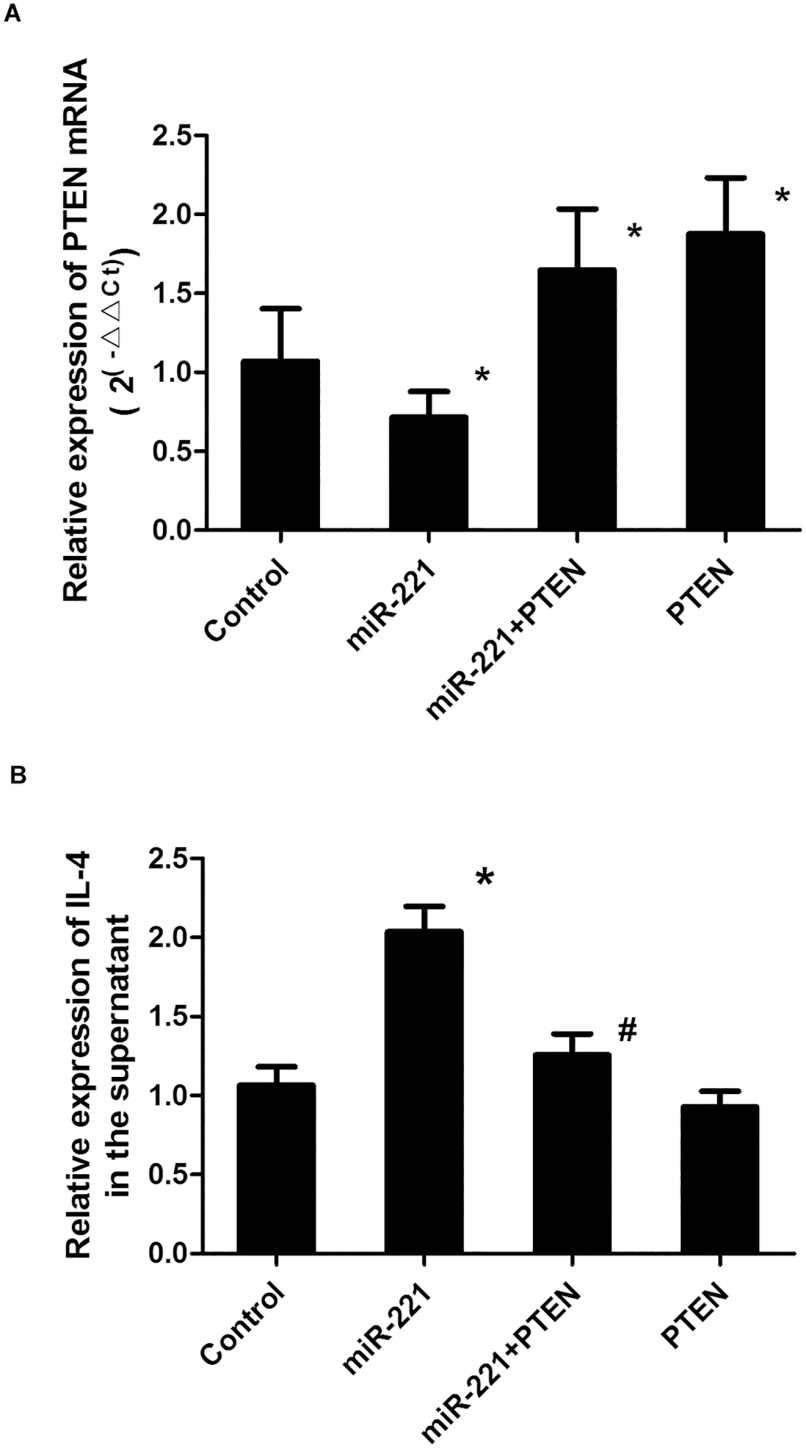
Effects of PTEN overexpression on *miR-221-*induced IL-4 secretion. After transfection with the PTEN overexpression lentivirus vector in cells overexpressing *miR-221*, *PTEN* mRNA levels (A) and IL-4 concentrations in the supernatant (B) were measured. *: *P* < 0.05 compared with the control group; #: *P* < 0.05 compared with the control group (n = 6).

To further illustrate the effects of PTEN on IL-4 secretion in *miR-221*-stimulated P815 cells, P815 cells were transfected with a PTEN overexpression lentivirus vector in cells overexpressing *miR-221*. PCR analysis showed that PTEN lentivirus transfection increased the expression of *PTEN* mRNA (*P* < 0.05; Fig 4A), demonstrating successful transfection. Moreover, in cells overexpressing PTEN, the IL-4 concentration was decreased compared with that in cells overexpressing *miR-221* alone, suggesting that the positive effects of *miR-221* on IL-4 secretion in mast cells could be reversed by PTEN (*P* < 0.05; Fig 4B).

### TLR expression and p38 phosphorylation increased in cells overexpressing *miR-221*

**Fig 5:**
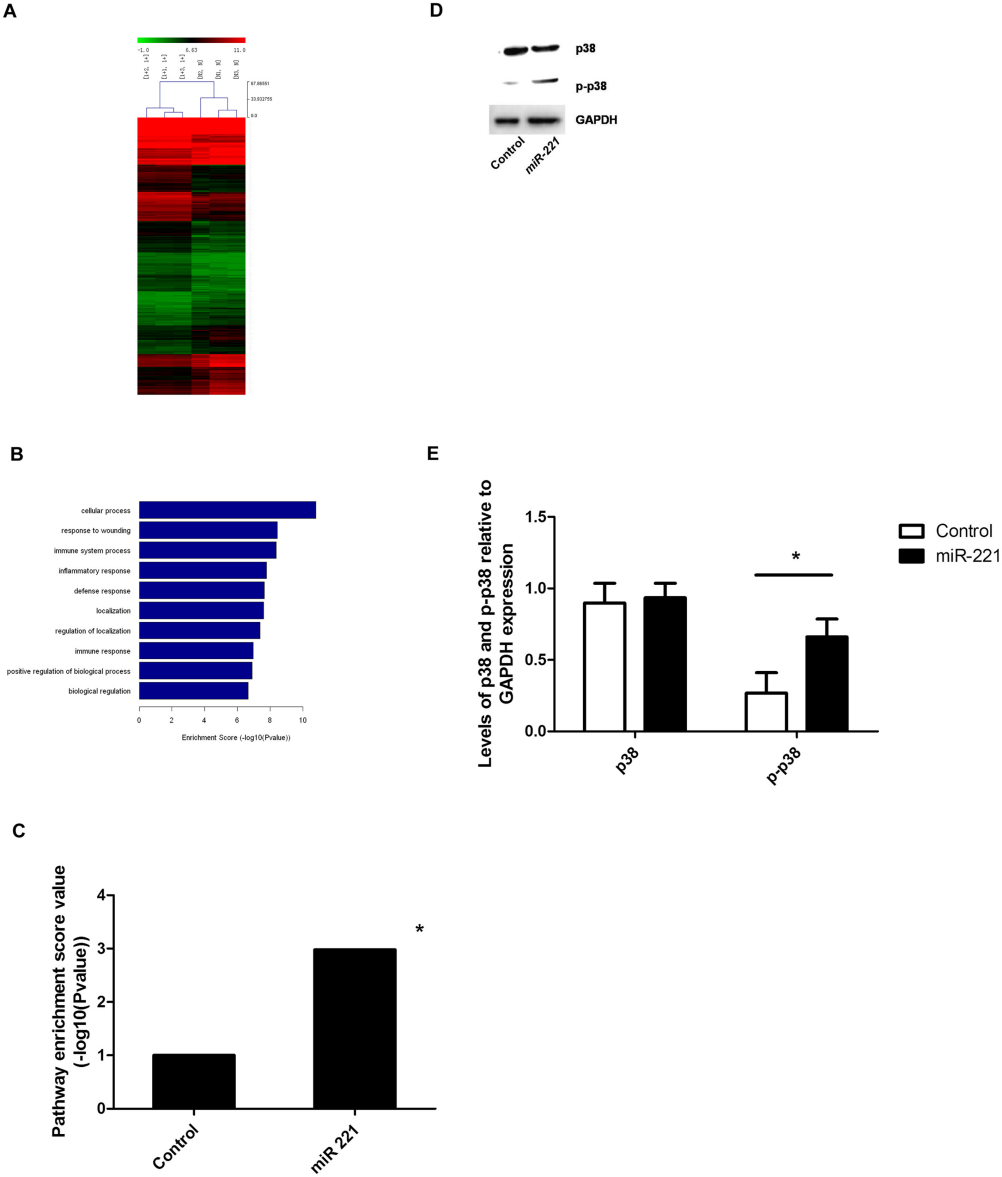
Changes in p38 expression and phosphorylation in response to *miR-221* overexpression. A) Heat map of differentially expressed genes between cells overexpressing *miR-221* and control cells (1+: *miR-221*; N+: control; *P* < 0.05; n = 3). B) Gene ontology analysis. *P* < 0.05 (n = 3). C) Analysis of the expression of TLR pathway components in cells overexpressing *miR-221* and control cells. *: *P* < 0.05 (n = 3). D, E) Total p38 protein expression and p38 phosphorylation were analyzed in control cells and cells overexpressing *miR-221*. *: *P* < 0.05 (n = 3 experiments).

To explore changes in signaling pathway components in cells overexpressing *miR-221*, we used gene chip analysis. As shown in Fig 5A, there were dramatic differences in overall gene expression in the context of *miR-221* overexpression; these changes were primarily related to the inflammation reaction category in gene ontology (GO) analysis. Among the differentially expressed genes, targets in the TLR signaling pathway, including TLR-1, -4, -6, and -7, were upregulated (*P* = 0.001). We focused on p38, a protein found downstream of the TLR pathway that has been reported to be modulated by PTEN [10]. While p38 protein levels did not change significantly after overexpression of *miR-221*, the phosphorylation of p38 increased (*P* < 0.05; Fig 5D and 5E).

### Treatment with a p38 pathway inhibitor blocked the secretion of IL-4

**Fig 6:**
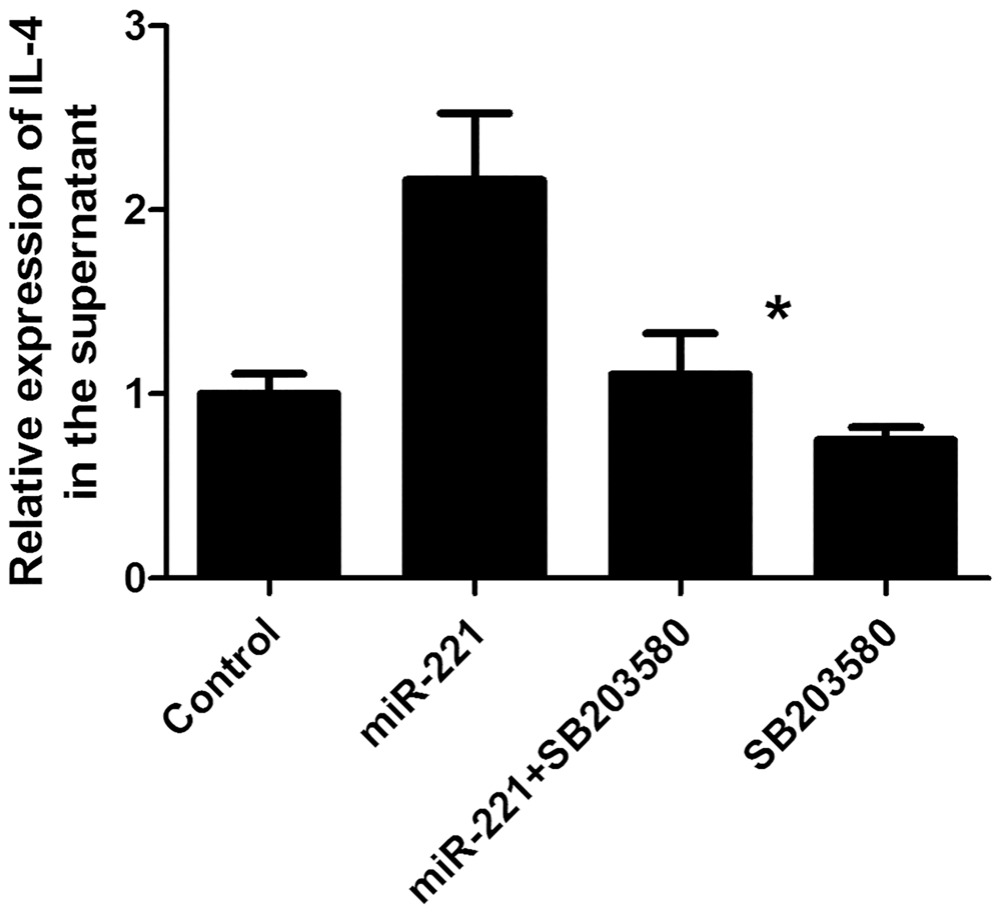
Effects of p38 inhibition on *miR-221*-induced IL-4 secretion. Cells overexpressing *miR-221* were treated with the PI3K/Akt inhibitor SB203580, and the concentration of IL-4 in the supernatant was measured. *: *P* < 0.05 compared with untreated cells overexpressing *miR-221* (n = 6).

To further explore the role of p38 in the regulation of IL-4 in the TLR signaling pathway, cells overexpressing *miR-221* were treated with a specific p38 MAPK signaling pathway inhibitor (SB203580), and the IL-4 concentration in the supernatant was measured. After treatment with SB203580, IL-4 levels in the supernatant were decreased compared with that in untreated cells overexpressing *miR-221* (*P* < 0.05). In contrast, no differences were observed in control cells, indicating that *miR-221* could activate p38 protein, increase p38 phosphorylation, and thereby regulate the release of IL-4 (Fig 6).

### NF-κB activity increased in cells overexpressing *miR-221*, and inhibition of NF-κB suppressed *miR-221*-induced IL-4 secretion

**Fig 7:**
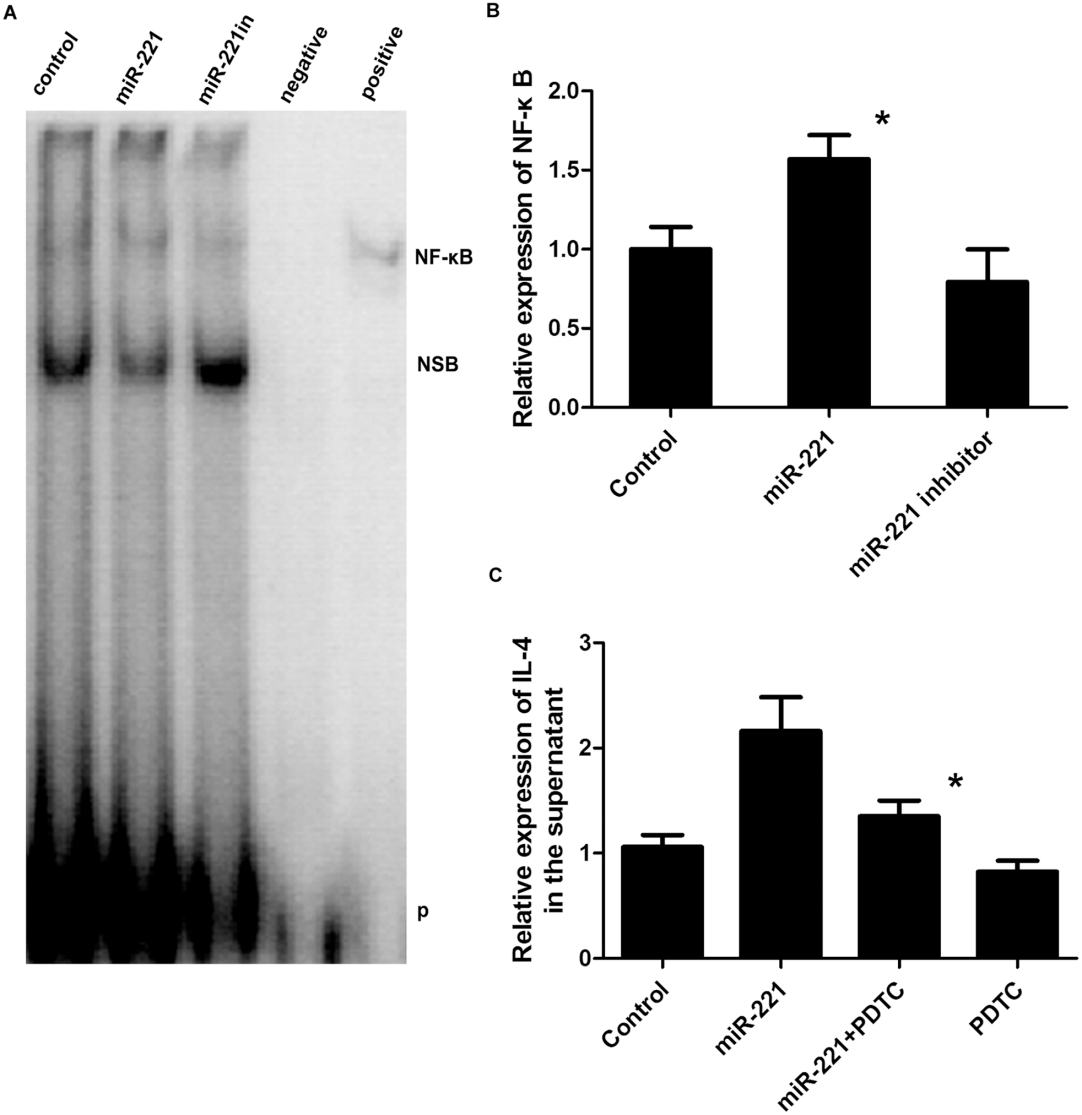
Changes in NF-κB expression in response to different *miR-221* levels and effects of NF-κB inhibition on *miR-221-*induced IL-4 secretion. A, B) Analysis of NF-κB DNA binding activity by EMSA. Nuclear proteins were extracted before detection. *: *P* < 0.05 compared with the control group (n = 3 experiments). C) Effects of PDTC treatment on the levels of IL-4 in the supernatant. *: *P* < 0.05 (n = 6). NSB: nonspecific binding; P: free biotin-labeled probe.

Many studies have demonstrated that a variety of inflammatory processes are regulated through nuclear factor NF-κB signaling. Therefore, we next examined NF-κB protein expression by EMSA using nuclear extracts from cells exhibiting upregulation or downregulation of *miR-221*. As shown in Fig 7A and 7B, the levels of NF-κB DNA binding in nuclear fractions were higher in cells overexpressing *miR-221* compared with those in control cells, suggesting that *miR-221* induced the activation of NF-κB. In contrast, there was no significant difference in cells exhibiting *miR-221* downregulation. To investigate the involvement of NF-κB in *miR-221*-induced IL-4 production, cells overexpressing *miR-221* were treated with PDTC, an inhibitor of the NF-κB pathway. Additionally, a decrease in IL-4 secretion was observed, indicating that *miR-221* could activate NF-κB to upregulate IL-4 in the supernatant (Fig 7C).

## Discussion

In this study, we used a murine asthma model to examine the role of *miR-221* in asthma. We observed increased total cell and eosinophil numbers in the murine asthma model, indicating the presence of inflammatory cell infiltration, a major pathological change in asthma. Moreover, *miR-221* was upregulated in the lungs of model mice, suggesting that *miR-221* may play an important role in the onset or development of asthma. Our results also provided important insights into the mechanisms through which *miR-221* regulates inflammation and signaling, involving the TLR, NF-κB, and MAPK pathways.

In this study, we found that *miR-221* increased IL-4 secretion from P815 mouse mast cells. Existing studies have suggested that mast cells may play an important role in immediate and delayed reactions in bronchial asthma [2, 11, 12], and that IL-4 is synthetized and secreted by mast cells [13]. Additionally, an imbalance in the T helper cell (Th) l/Th2 ratio and a predominance of Th2 cells in the airways are important for the pathogenesis of asthma [14]. IL-4 is a strong regulatory factor involved in the differentiation of Th2 cells, promotes synthesis and secretion of IgE from B lymphocytes, and increases the expression of the IgE Fc low-affinity receptor. Therefore, in our study, IL-4 was used as an index of mast cell secretion.

*miR-221* is located on the X chromosome and is important for cell proliferation, differentiation, and apoptosis, especially in tumors. The expression of *miR-221* in tumor tissues from patients with breast cancer, prostate cancer, and bladder cancer is much higher than that in normal tissues [15]. Moreover, *miR-221* has been suggested to function as a biomarker for the early diagnosis of cancer. Another study found that *miR-221* is involved in the regulation of inflammatory reactions. In cholangitis, *miR-221* regulates the secretion of cell adhesion molecule 1 induced by interferon gamma in bile duct cells [16]. The expression of *miR-221* was higher in the lung tissues of asthmatic mice than in those of normal mice, and analysis of lung pathology showed that inflammatory cell infiltration was increased. In addition, miRNAs have been reported to regulate mast cell secretion. Specifically, *miR-221* can modulate the mast cell cycle by inhibiting p27Kip1 [17]. In our previous work, we found that LPS stimulation increases IL-4 secretion from P815 cells. Similarly, in this study, LPS stimulation promoted *miR-221* expression in P815 cells, and changes in *miR-221* expression altered IL-4 secretion in P815 cells. These results suggested that *miR-221* could regulate the secretory function of mast cells.

Our analysis identified *PTEN* as a target of *miR-221*. Moreover, deletion of PTEN reversed the effects of *miR-221* stimulation in P815 cells. For example, downregulation of PTEN increased IL-4 secretion, whereas overexpression of PTEN blocked IL-4 secretion. These results demonstrated that PTEN could reverse the effects of overexpression of *miR-221* on secretion of IL-4 in mast cells. PTEN has been reported to have an important role in cell differentiation, senescence, and apoptosis. Recently, inhibition of PTEN expression in macrophages was shown to promote the release of a large number of inflammatory mediators [18]. Additionally, in allergic inflammatory responses, the *PTEN* gene may inhibit the apoptosis and chemotaxis of eosinophils [19]. Result in our study on the role of PTEN is consistent with the previous studies.

A gene expression microarray was then used to further elucidate the signaling pathways involved in the regulation of *miR-221* in P815 cells. In P815 cells overexpressing *miR-221*, TLR-1, -4, -6, and -7 expression increased. TLRs are important in innate immunity and adaptive immunity and play an important role in tumors, allergic diseases, and cardiovascular diseases [20, 21]. Some recent studies have suggested that there may be a link between mast cells and TLRs in asthma [22, 23, 24]. TLRs can identify LPS or related ligands to activate transcription factors, such as MAPK, and can promote the secretion of inflammatory cytokines and chemokines. Previous studies have identified three parallel MAPK signaling pathways, i.e., extracellular signal-regulated kinase (ERK), c-Jun N-terminal kinase (JNK)/SAPK, and p38 MAPK. p38MAPK participates in inflammation and has been reported to be modulated by PTEN [10], and PTEN was known as a non-specific supressor of cell signaling pathways, including MAPK; therefore, p38 was analyzed in this study. Western blot analysis showed that phosphorylation of p38 increased in P815 cells overexpressing *miR-221*. Moreover, inhibition of p38 MAPK by SB203580 treatment blocked the secretion of IL-4. This suggested that *miR-221* may increase the secretion of IL-4 by phosphorylating and activating p38 protein.

The role for p38 MAPK activity has been proved in the generation of proinflammatory cytokines by regulating the activation of NF-κB. Our data also suggested that NF-κB activity was increased in response to overexpression of *miR-221*, resulting in induction of IL-4 in P815 cells. NF-κB has been shown to activate inflammatory responses, and activation of p38 leads to NF-κB transcriptional activation. Consistent with this and our other findings, inhibition of NF-κB in cells overexpressing *miR-221* resulted in decreased IL-4 secretion, suggesting that *miR-221* may increase the secretion of IL-4 through activation of the NF-κB pathway.

In conclusion, *miR-221* expression was increased in a model of asthma in mice. Additionally, stimulation of P815 mouse mast cells with LPS increased *miR-221* expression, and modulation of *miR-221* expression altered IL-4 secretion in P815 cells. PTEN, a target of *miR-221* regulation, and the p38/NF-κB pathway were involved in the regulation of mast cells by *miR-221*. This may help to further understand the mechanism of asthma.

## Supporting Information

S1 Data Data for studies. (DOC)
