# Supplementary material for: miRNA-221-3p Enhances the Secretion of Interleukin-4 in Mast Cells through the Phosphatase and Tensin Homolog/p38/Nuclear Factor-kappaB Pathway
Source: PLoS One. 2016 Feb 22;11(2):e0148821. doi: 10.1371/journal.pone.0148821 (PMC4764704; doi:10.1371/journal.pone.0148821)
Supplement: S1 Data — (DOC) [file pone.0148821.s001.doc]

**S1 Data. Data for Fig. 1A**

| Total cells(×10^5^) | | Eosinophils(×10^5^) | |
| --- | --- | --- | --- |
| Normal | Asthma model | Normal | Asthma model |
| 5.9 | 11.9 | 0.4 | 4.3 |
| 5.5 | 9.3 | 0.3 | 8.8 |
| 8.8 | 15.2 | 0.4 | 3.8 |
| 5.3 | 14.9 | 2.0 | 9.3 |
| 4.2 | 12.4 | 0.3 | 10.0 |
| 8.1 | 13.8 | 0.5 | 3.9 |
| 3.7 | 9.8 | 2.6 | 4.7 |
| 3.2 | 15.3 | 0.2 | 7.9 |

**S2 Data. Data for Fig. 1B**

| Normal | Asthma model |
| --- | --- |
| 0.87 | 2.79 |
| 1.42 | 1.99 |
| 1.37 | 3.57 |
| 0.75 | 2.7 |
| 0.85 | 2.22 |
| 1.66 | 3.41 |
| 0.73 | 3.76 |
| 0.54 | 2.41 |

**S3 Data. Data for Fig. 2A**

| Control | LPS |
| --- | --- |
| 0.98 | 1.32 |
| 0.87 | 1.21 |
| 0.94 | 1.14 |
| 0.92 | 1.23 |
| 1.08 | 1.41 |
| 0.81 | 1.30 |

**S4 Data. Data for Fig. 2B**

| Control | LV3NC | miR-221 |
| --- | --- | --- |
| 1.412 | 0.912 | 5.962 |
| 1.184 | 0.846 | 6.334 |
| 0.797 | 0.725 | 6.137 |
| 0.957 | 1.021 | 5.098 |
| 1.469 | 1.228 | 4.836 |
| 1.094 | 0.952 | 5.357 |

**S5 Data. Data for Fig. 2C**

| Control | miR 221 | miR 221 inhibitor | Negative control |
| --- | --- | --- | --- |
| 1.00 | 1.75 | 1.56 | 0.37 |
| 1.25 | 2.29 | 0.88 | 0.73 |
| 1.13 | 2.73 | 1.38 | 0.48 |
| 0.88 | 2.38 | 1.06 | 0.62 |
| 0.69 | 2.48 | 0.75 | 0.25 |
| 1.17 | 1.65 | 0.54 | 0.75 |

**S6 Data. Data for Fig.** 3B

|  | PTEN |
| --- | --- |
| Control | 0.99 |
|  | 0.87 |
|  | 0.74 |
| miR-221 | 0.57 |
|  | 0.53 |
|  | 0.23 |

**S7 Data. Data for Fig.** 4A

| PTEN | Control | miR-221 | miR-221+PTEN |
| --- | --- | --- | --- |
| 1.34 | 0.61 | 0.56 | 1.73 |
| 2.22 | 1.39 | 0.68 | 1.05 |
| 1.70 | 0.89 | 0.98 | 1.35 |
| 2.07 | 1.29 | 0.83 | 1.98 |
| 1.69 | 0.84 | 0.56 | 1.74 |
| 2.24 | 1.40 | 0.68 | 2.05 |

**S8 Data. Data for Fig.** 4B

| Control | miR-221 | miR-221+PTEN | PTEN |
| --- | --- | --- | --- |
| 1.00 | 2.11 | 1.40 | 0.98 |
| 1.18 | 1.84 | 1.36 | 0.86 |
| 1.13 | 1.90 | 1.18 | 1.08 |
| 0.97 | 2.26 | 1.04 | 0.88 |
| 0.92 | 1.95 | 1.32 | 0.96 |
| 1.20 | 2.15 | 1.25 | 0.80 |

**S9 Data. Data for Fig. 5C (1)**

| PathwayID | Definition | Fisher-Pvalue | Enrichment_Score |
| --- | --- | --- | --- |
| mmu04620 | Toll-like receptor signaling pathway - Mus musculus | 0.001 | 2.978 |

**S10 Data. Data for Fig. 5C (2)**

| Group | Raw Intensities | | | |
| --- | --- | --- | --- | --- |
|  | Tlr1 | Tlr4 | Tlr6 | Tlr7 |
| Control | 5.00 | 57.84 | 168.44 | 375.53 |
|  | 5.00 | 44.87 | 99.37 | 396.39 |
|  | 5.00 | 58.97 | 131.73 | 320.95 |
| miR-221 | 17.75 | 194.95 | 578.89 | 726.96 |
|  | 16.15 | 151.85 | 492.28 | 390.50 |
|  | 11.90 | 167.10 | 487.86 | 534.32 |

**S11 Data. Data for Fig.** 5E

|  | P38 | P-P38 |
| --- | --- | --- |
| Control | 0.77 | 0.35 |
|  | 0.89 | 0.1 |
|  | 1.04 | 0.35 |
| miR-221 | 1.01 | 0.83 |
|  | 0.97 | 0.62 |
|  | 0.82 | 0.53 |

**S12 Data. Data for Fig. 6**

| Control | miR-221 | miR-221+SB203580 | SB203580 |
| --- | --- | --- | --- |
| 1.00 | 1.83 | 1.18 | 0.42 |
| 1.03 | 1.76 | 1.12 | 0.62 |
| 0.96 | 2.09 | 1.11 | 0.40 |
| 1.20 | 2.00 | 1.24 | 0.53 |
| 1.15 | 2.40 | 1.03 | 0.44 |
| 0.97 | 2.34 | 0.90 | 0.51 |

**S13 Data. Data for Fig. 7B**

| Control | miR-221 | miR-221 inhibitor |
| --- | --- | --- |
| 15.82 | 24.24 | 13.78 |
| 14.36 | 19.13 | 11.94 |
| 11.16 | 21.49 | 7.05 |

**S14 Data. Data for Fig. 7C**

| Control | miR-221 | miR-221+PDTC | PDTC |
| --- | --- | --- | --- |
| 1 | 2.441 | 1.112 | 0.827 |
| 0.975 | 1.723 | 1.323 | 0.948 |
| 0.988 | 2.519 | 1.523 | 0.727 |
| 1.088 | 2.042 | 1.375 | 0.926 |
| 1.281 | 1.894 | 1.487 | 0.829 |
| 1.023 | 2.348 | 1.294 | 0.688 |
